# Genetic diversity and population structure of *Prunus mira* (Koehne) from the Tibet plateau in China and recommended conservation strategies

**DOI:** 10.1371/journal.pone.0188685

**Published:** 2017-11-29

**Authors:** Wenquan Bao, Tana Wuyun, Tiezhu Li, Huimin Liu, Zhongmao Jiang, Xuchun Zhu, Hongyan Du, Yu-e Bai

**Affiliations:** 1 Non-Timber Forest Research and Development Center, Chinese Academy of Forestry, Zhengzhou, Henan, People’s Republic of China; 2 College of Forestry, Inner Mongolia Agricultural University, Hohhot, People’s Republic of China; Consiglio Nazionale delle Ricerche, ITALY

## Abstract

*Prunus mira* Koehne, an important economic fruit crop with high breeding and medicinal values, and an ancestral species of many cultivated peach species, has recently been declared an endangered species. However, basic information about genetic diversity, population structure, and morphological variation is still limited for this species. In this study, we sampled 420 *P*. *mira* individuals from 21 wild populations in the Tibet plateau to conduct a comprehensive analysis of genetic and morphological characteristics. The results of molecular analyses based on simple sequence repeat (SSR) markers indicated moderate genetic diversity and inbreeding (*A* = 3.8, *Ae* = 2.5, *He* = 0.52, *Ho* = 0.44, *I* = 0.95, *F*_*IS*_ = 0.17) within *P*. *mira* populations. STRUCTURE, GENELAND, and phylogenetic analyses assigned the 21 populations to three genetic clusters that were moderately correlated with geographic altitudes, and this may have resulted from significantly different climatic and environmental factors at different altitudinal ranges. Significant isolation-by-distance was detected across the entire distribution of *P*. *mira* populations, but geographic altitude might have more significant effects on genetic structure than geographic distance in partial small-scale areas. Furthermore, clear genetic structure, high genetic differentiation, and restricted gene flow were detected between pairwise populations from different geographic groups, indicating that geographic barriers and genetic drift have significant effects on *P*. *mira* populations. Analyses of molecular variance based on the SSR markers indicated high variation (83.7% and 81.7%), whereas morphological analyses revealed low variation (1.30%–36.17%) within the populations. Large and heavy fruits were better adapted than light fruits and nutlets to poor climate and environmental conditions at high altitudes. Based on the results of molecular and morphological analyses, we classified the area into three conservation units and proposed several conservation strategies for wild *P*. *mira* populations in the Tibet plateau.

## Introduction

*Prunus mira* Koehne (2n = 2x = 16) is a perennial woody plant that belongs to the genus *Prunus* of the family Rosaceae [1]. It is native to China, and it is an ancestral species of many cultivated peach species, including *P*. *davidiana*, *P*. *kansuensis*, and *P*. *ferganensis* [2–4]. *P*. *mira* is also considered a valuable “living fossil” of peach species because of its long life cycle (over 1000 years). Its fruits are rich in nutrients (vitamin C, calcium, and ferrum), fatty acids (oleic acid, linoleic acid, cetylic acid, and octadecanoic acid), and active medicinal ingredients (arbutin). Therefore, in addition to supplying fruits, *P*. *mira* is used in Chinese traditional medicine to treat irregular menses, fractures, and congestion [5]. Considering that *P*. *mira* has high tolerance to environmental stress (drought, cold, and barren soil) and high yields, it has great potential with regard to peach breeding [6,7].

*P*. *mira* is widely distributed along the Yarlung Zangbo Grand Canyon and its tributary basins (Parlung Zangbo Basin and Nyang River Basin) in the Tibet plateau [1,8]. Shannan and Linzhi, located in the middle and lower reaches of the Yarlung Zangbo Grand Canyon, are the two areas where *P*. *mira* densely inhabits [3,7]. Shanan is characterized by a plateau temperate arid climate, with a mean altitude of 3700 m. Linzhi has a tropical humid and semi-humid climate, with a mean altitude of 3000 m. Owing to different climates and landscapes, the *P*. *mira* wild resources from different areas develop different phenological phases [9,10]. For instance, the flowering time of *P*. *mira* is March 15–25 in Shannan and March 9–20 in Linzhi. Recent field investigations revealed that morphological characteristics such as fruit size, nutlet size, and nutlet surfaces, were considerably different between *P*. *mira* wild resources in Shannan and those in Linzhi. Previous studies [7,8,11,12] revealed high genetic and morphological variations within wild *P*. *mira* resources, as well as many excellent breeding resources in the Shannan and Linzhi areas. Unfortunately, because of deforestation, over-harvest, and road building, the natural distribution and population size of *P*. *mira* have been remarkably reduced [5,6]. Although several studies have shown genetic diversity and morphological variations in *P*. *mira* [6–8], they were conducted using a small number of individuals from a small-scale district, and the results provided very limited information on genetic and morphological variations in this species. A comprehensive study of *P*. *mira* genetic and morphological variations is still lacking. The knowledge of genetic and morphological variations can reveal the evolutionary history and existing situations associated with a plant species [13–15]. Therefor, studying the genetic diversity, population structure, and morphological variation of *P*. *mira* is crucial for the protection of this endangered species. For decades, numerous molecular markers such as single nucleotide polymorphisms (SNP), random amplified polymorphisms DNA (RAPDs), simple sequence repeats (SSRs), and inter-simple sequence repeats (ISSRs), have been developed to analyze the genetic diversity and population structure of plant species [16–19]. Among these markers, SSRs have been the first choice for studies of genetic diversity and population structure, because of their excellent characteristics, including high polymorphism, co-dominant inheritance, and low inquiry of DNA [20]. Furthermore, SSRs were found to be highly transferable among *Prunus* species [21,22]. Therefore, numerous SSRs that were developed in related species can be effective tools for conducting genetic analysis of *P*. *mira*. Morphological classification is a traditional and intuitive means of characterizing plant species, and it has been widely used to evaluate morphological variation and differentiation within and among populations [23]. The same data can be used to conduct various types of morphological analyses to provide valuable information on morphological variations for plant conservation.

Therefore, in this study, we used 25 SSR markers and 11 morphological characteristics to assess the genetic diversity, population structure, and morphological variation of 21 wild *P*. *mira* populations that were collected from the Tibetan plateau. This study aimed to (1) comprehensively reveal the genetic diversity and population structure of *P*. *mira*; (2) estimate morphological variation among and within populations; and (3) propose effective conservation strategies for the studied populations.

## Materials and methods

### Materials

The leaf samples, fruits, and nutlets of 420 individuals were collected from 21 wild *P*. *mira* populations along the Yarlung Zangbo Grand Canyon and its tributary basin in the Tibet plateau (S1 Fig, Table 1). The longitude, latitude, and altitude of each individual were detected using a global position system. For molecular analyses, 20 individuals were sampled from each population, with a minimum distance of 50 m between any two individuals. Healthy and young leaves were collected and immediately preserved with silica gel for DNA extraction. For morphological analyses, 20 fruits and 20 nutlets were randomly selected from each individual. Finally, 8400 fruits and 8400 nutlets were collected to analyze the morphological variation within and among *P*. *mira* populations.

**Table 1:** Location and sample size of the 21 populations of *Prunus mira* Koehne used in this study.

Population	Population ID	Sample size	Latitude (N)	Longitude (E)	Altitude (m)	Habitats
Yusong, Tibet	P1	20	29°33′–29°37′	96°19′–96°22′	2921–2958	Valley
Bengga, Tibet	P2	20	29°34′–29°51′	96°50′–96°54′	2931–2962	Valley
Zhongsha, Tibet	P3	20	29°35′–29°54′	95°44′–95°56′	2939–2972	Valley
Danniang, Tibet	P4	20	29°43′–29°58′	95°23′–95°36′	2944–2963	Valley
Langga, Tibet	P5	20	30°01′–30°36′	95°35′–95°49′	2937–2966	Hillside terraces
Luxia, Tibet	P6	20	30°06′–30°11′	95°09′–95°13′	3232–3244	Roadside
Jieguo, Tibet	P7	20	30°01′–30°59′	94°47′–94°56′	3198–3228	Around rural house
Qiangna, Tibet	P8	20	29°45′–29°53′	94°34′–94°59′	3295–3311	High slope
Caiba, Tibet	P9	20	29°57′–30°05′	94°24′–94°57′	3286–3302	Around rural house
Bujiu, Tibet	P10	20	29°16′–29°50′	94°04′–94°22′	3129–3169	Roadside
Duodang, Tibet	P11	20	29°43′–29°49′	94°18′–94°26′	3238–3256	Hillside terraces
Gongzhong, Tibet	P12	20	29°30′–29°44′	94°32′–94°39′	3111–3152	Around rural house
Baga, Tibet	P13	20	29°49′–29°55′	93°56′–94°04′	3294–3338	Roadside
Gangga, Tibet	P14	20	29°20′–29°29′	94°16′–94°24′	2953–2979	Hillside terraces
Zhuomu, Tibet	P15	20	29°16′–29°24′	93°13′–93°53′	3359–3394	Roadside
Taohua, Tibet	P16	20	29°54′–30°01′	93°30′–93°41′	3297–3318	High slope
Xiake, Tibet	P17	20	29°31′–29°39′	92°43′–92°54′	3661–3703	Hillside terraces
Semai3, Tibet	P18	20	29°15′–29°23′	92°30′–92°39′	3778–3793	Valley
Semai6, Tibet	P19	20	29°22′–29°30′	92°00′–92°59′	3836–3844	Valley
Taohua2, Tibet	P20	20	29°25′–29°31′	91°18′–91°32′	3802–3839	High slope
Tunmi, Tibet	P21	20	29°08′–29°22′	92°05′–92°17′	3667–3708	Hillside terraces

In this study, the 21 sampled populations covered the entire distribution of *P*. *mira* in the Tibet plateau. Among these populations, P1, P2, P3, P4, and P5 were located in Yarlung Zangbo Grand Canyon National Nature Reserve, and P14 was distributed in the Qomolangma National Nature Reserve. Our study was permitted and approved by these authorities. Furthermore, P8, P11, P16, P17, P18, P19, P20, and P21 were distributed in the wild, and P6, P10, P13, and P15 were distributed around roadsides, which were taken for the wild species. No specific permits were required for collecting these samples from the wild, and no specific permissions were required for these locations or activities. Furthermore, P7, P9, and P12 were distributed around rural houses, and were collected with the permission of private land owners. No endangered or protected species were sampled. All populations could be divided into two groups according to their geographic distribution. The Linzhi group (G1) contained 16 populations (P1–P16). Among these populations, P1, P2, P3, P4, P5, and P14 were distributed in low-altitude areas (2921–2979 m), and P6, P7, P8, P9, P10, P11, P12, P13, P15, and P16 were located in medium-altitude areas (3111–3394 m; Table 1). The Shannan group (G2) included the five remaining populations (P17–P21), which were distributed in the high-altitude areas (3661–3844 m).

### DNA concentration, SSR analysis, and polymerase chain reaction amplification

The total genomic DNA of all dried leaves was extracted using the CTAB method [24], and DNA concentration and quality were tested using a 1% agarose gel. In all, 25 microsatellite markers, developed from three species related to *P*. *mira*, were selected to determine the genetic diversity and population structure of *P*. *mira*, including the 22 loci from *P*. *persica* [25–30], one locus from *P*. *cerasus* [31], and two loci from *P*. *avium* [32] (S1 Table). The forward primers of all SSR markers were assembled using M13 (5′-TGTAAAACGACGGCCAGT-3′), which contained one of four fluorescent dyes—FAM, NED, VIC, and PET. Polymerase chain reaction (PCR) amplification was conducted in a 20 μL reaction volume that contained 20 ng DNA, 0.2 μL 2× Tab Master Mix, 2 μL 10× buffer, 0.5 μL dNTP (0.25 mol·L^-1^), 0.5 μL 10% DMSO, 14.8 μL ddH_2_O, and 0.5 μL of each primer (0.8 μmol·L^-1^). The PCR protocol was as follows: 95°C for 5 min; followed by 30 cycles of 95°C for 30 s, 55°C for 30 s, and 72°C for 30 s; and a final extension at 72°C for 5 min. PCR products were analyzed using an ABI 3500XL (Applied Biosystems, USA) DNA Analyzer. The amplicon fragments were sized using Gene-Marker (Soft Genetics LLC, USA), and the details are shown in S2 Table.

### Genetic analysis

GenAlEx version 6.501 [33] was used to convert size data into various formats for genetic analysis. Based on the Bayesian method, we used STRUCTURE version 2.3.4 [34] to determine the population structure of the 21 *P*. *mira* populations. Twenty independent runs were performed for each set, with a K value between 1 and 21, length of burn-in period of 1 × 10^5^ iterations, and 1 × 10^5^ MCMC iterations. No prior information about populations was used. The method described by Evanno et al. [35] was used to calculate the distribution of delta K (ΔK) using the online website STRUCTURE HARVESTER [36] (http://taylor0.biology.ucla.edu/struct_harvest/). Permutations of the most likely results among all runs for each K were performed in CLUMPP [37], and the final figure was visualized using Distruct software [38].

GENELAND [39,40] is a powerful way to detect genetic boundaries in R (2011). This method was used to estimate the number of clusters and their spatial patterns. The analysis was based on an uncorrelated frequency model, and was conducted over 10 replicates for each K value (1–10). The null allele models were selected to infer the number of clusters using 1,000,000 MCMC iterations, of which every 1000^th^ was retained. The replicates with the highest mean logarithm of posterior probability were used to compute the posterior probabilities of population membership for each pixel of the spatial domain with a burn-in of 500.

*P*. *mira* genetic variation was estimated by conducting an analysis of molecular variance (AMOVA) in Arlequin version 3.5 [41]. This analysis subdivided all individuals into 21 populations and K genetic clusters. Three hierarchical divisions were identified based on the genetic variance: within populations, among populations within genetic clusters, and among genetic clusters using a nonparametric permutation procedure that incorporated 10,000 iterations. An unrooted unweighted pair-group method with arithmetic means (UPGMA) tree was constructed using Nei’s genetic distance [42] in NTSYS PC version 2.10 [43]. Mantel tests were conducted between the genetic distance (*F*_*ST*_/(1—*F*_*ST*_)) and geographic distance (km) in GenAlEx version 6.501.

GenAlEx version 6.501 was used to calculate the following genetic indices for loci, populations, and genetic clusters identified in the STRUCTURE analysis: allele number (*A*), effective allele number (*Ae*), private allele number (*Np*), Shannon’s information index (*I*), expected heterozygosity (*He*), observed heterozygosity (*Ho*), gene flow (*Nm*), and *F*-statistics indices (*F*_*IS*_, *F*_*IT*_, and *F*_*ST*_). The Hardy-Weinberg equilibrium (*HWE*) was tested for each locus and population using Arlequin version 3.1 with 100,000,000 steps in the Markov chain and 100,000 dememorization steps. Polymorphism information content (*PIC*) was calculated using the following formula [44]:

$$PIC=1-\sum_{i=1}^{n}P_{i}^{2}-\sum_{i=1}^{n-1}\sum_{j=i+1}^{n}2P_{i}^{2}P_{j}^{2}$$

where *P*_*i*_ and *P*_*j*_ are the frequency of the amplified alleles, and *n* is the number of alleles at each SSR locus.

### Morphologic analysis

In all, 20 fruits and 20 nutlets were randomly selected per individual to measure and calculate the following 11 morphological characteristics: fruit weight, fruit length, fruit width, fruit diameter, fruit shape index (fruit length/fruit width), nutlet weight, nutlet length, nutlet width, nutlet diameter, nutlet shape index (nutlet length/nutlet width), and nutlet surface. The weights of fruits and nutlets were tested using an electronic balance (accuracy of 0.001 g). The lengths, widths, and diameters of fruits and nutlets were measured using a digital Vernier caliper (accuracy of 0.001 mm). The mean values of all morphological characteristics were used to construct an unrooted UPGMA tree in NTSYS PC version 2.10 based on Nei’s genetic distance (1978). IBM SPSS Statistics 19 software was used to conduct Duncan’s multiple comparison analysis, calculate coefficients of variation (*CV*, %), and draw the boxplots of the 11 morphological characteristics. For clustering and statistical analyses, the three types of nutlet surfaces were classified as follows: 1 (smooth), 2 (shallow groove), and 3 (deep groove).

## Results

### Characteristics of SSR markers

Twenty-five SSR markers were selected to identify genotype 420 *P*. *mira* individuals. A total of 214 alleles were detected across all loci, ranging from four (UDP96-019) to 15 (BPPCT-025 and PMS67), with an average of 8.6 alleles per locus (S3 Table). The effective number of alleles (*Ae*) was between 1.5 (UDP96-019) and 7.5 (BPPCT-025), with an average of 3.6. The mean expected heterozygosity (*He*) was 0.52, and it exceeded the observed heterozygosity (*Ho* = 0.46). *F*-statistics showed moderate *F*_*ST*_ (0.15) and *F*_*IS*_ (0.13) values across the 25 loci, indicating moderate genetic differentiation across all sites. In addition, Shannon’s information index (*I*) ranged between 0.18 (Pchgms 4) and 2.07 (BPPCT-025), with an average of 1.19. The polymorphism information content (*PIC*) was between 0.82 (BPPCT-025) and 0.32 (Pchgms4), with an average of 0.62. Of the 25 SSR markers, six (UDP98-412, Pchgms4, BPPCT-004, UDP98-405, UDP98-408, and UDP96-019) were moderately informative (0.25 < *PIC* < 0.50), and 19 were highly informative (*PIC* > 0.50), indicating their high potential regarding the genetic diversity analysis of *P*. *mira* populations.

### Genetic structure and differentiation

**Fig 1:**
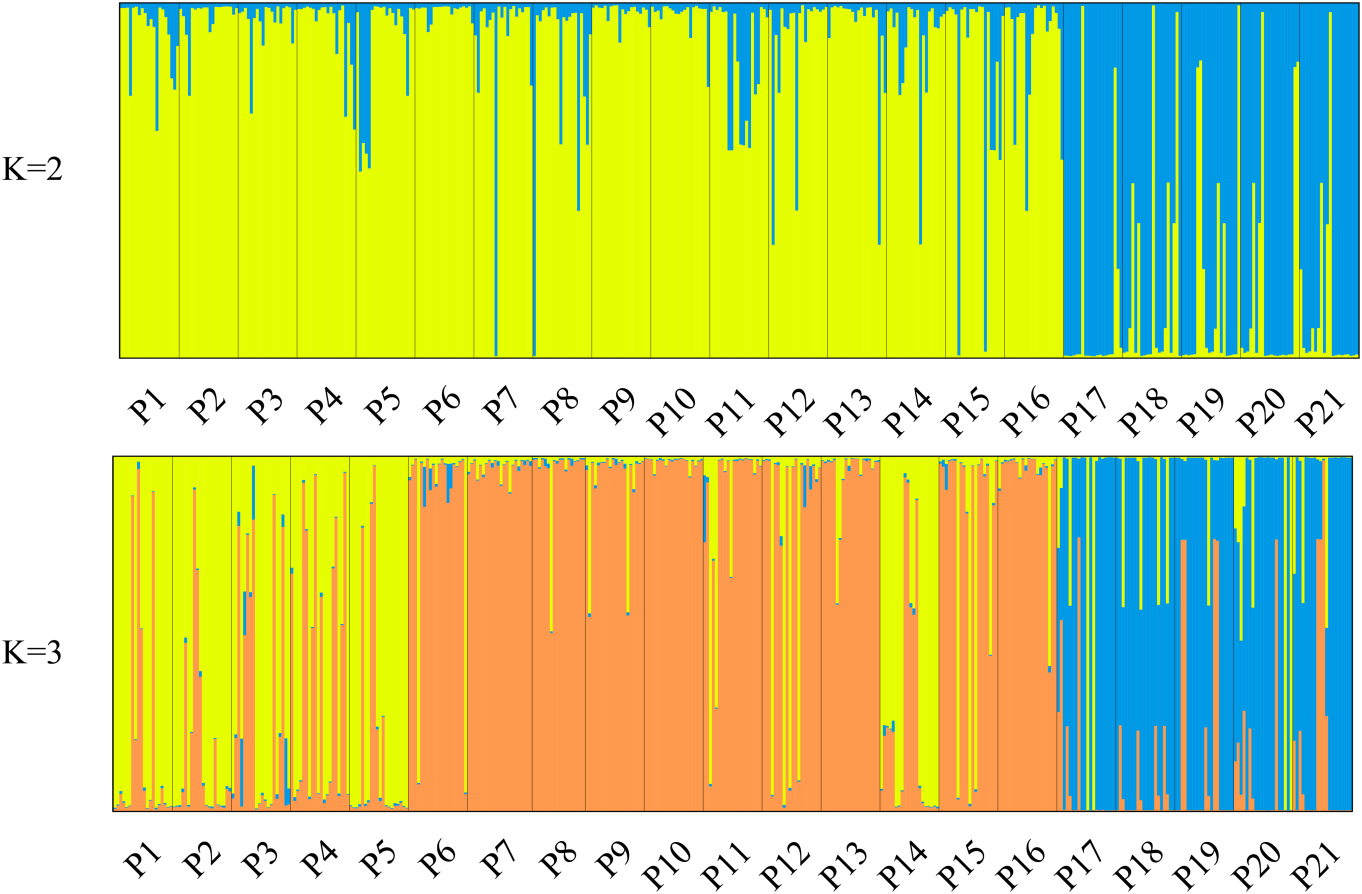
Clustering of 21 *P*. *mira* populations based on STRUCTURE analyses. Population structure of 21 *P*. *mira* populations at K = 2 and K = 3. Each individual is shown as a vertical line divided into segments representing the estimated membership proportion; different genetic clusters were inferred using STRUCTURE. At K = 2, high-altitude populations (P17–21) were separated from the medium- and low altitude populations. Furthermore, medium-altitude (P6–13, P15, P16) and low-altitude (P1–5, P14) populations were assigned to two distinct clusters at K = 3.

The genetic structure of 21 *P*. *mira* populations was tested using Bayesian clustering methods in STRUCTURE version 2.3.4. The classification of populations was highly correlated with their geographic altitudes. When K = 2, 16 low- and medium-altitude populations (P1–P16) from Linzhi group and five high-altitude populations (P17–P21) from Shannan group were assigned to two different clusters with high ancestry values (Q ≥ 0.80; Fig 1, S4 Table). When K = 3 and Q ≥ 0.60, 21 populations could be clearly assigned to three genetic clusters (Fig 1, S4 and S5 Tables). Cluster I consisted of six low-altitude populations (P1, P2, P3, P4, P5, and P14) from the Linzhi group. Of the 120 individuals from these populations, 88 individuals (73.33%) were assigned to Cluster I; 25 individuals (20.84%), to Cluster II; and seven individuals (5.83%) were mixed (Q < 0.60). Cluster II included 10 medium-altitude populations in the Linzhi group (P6, P7, P8, P9, P10, P11, P12, P13, P15, and P16). Among these populations, only 11 individuals (5.50%) were assigned to Cluster I, and the Q values of six individuals (3.00%) were below 0.60. Cluster III included five high-altitude populations (P17, P18, P19, P20, and P21) from the Shannan group. Among these populations, only 13 individuals (13.00%) were assigned to two other clusters, and 15 (15.00%) were mixed. The maximum value of ΔK was observed at K = 3 (S2 Fig), indicating that the 21 populations were potentially assigned to three clusters.

**Fig 2:**
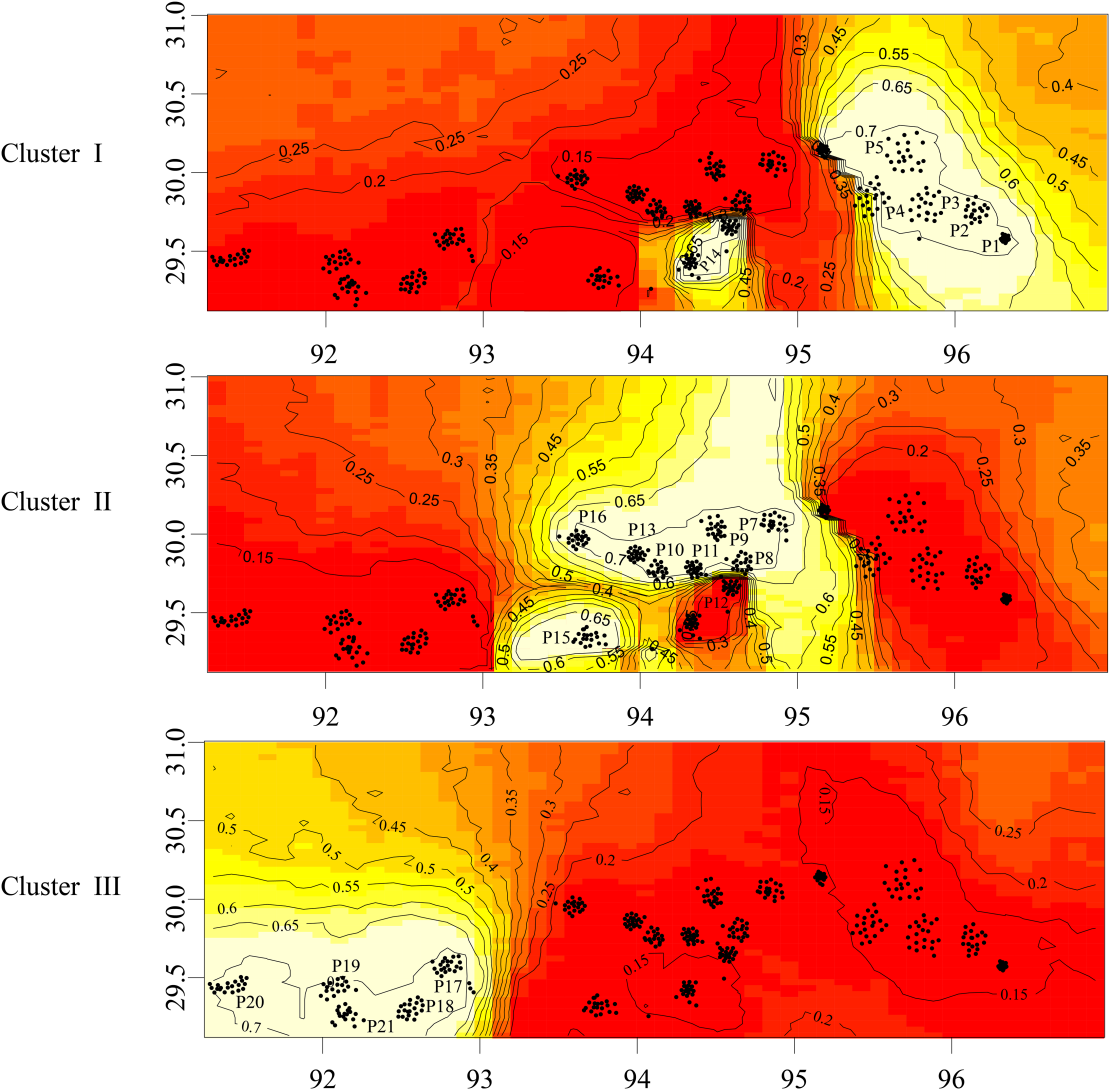
Clustering of 21 *P*. *mira* populations based on GENELAND analyses. Maps of posterior probabilities of population membership at K = 3 were inferred using GENELAND. The three maps show three clusters that were consistent with the three genetic clusters identified by the STRUCTURE analyses.

We further investigated the spatial patterns among the 21 *P*. *mira* populations based on an uncorrelated frequency model in GENELAND. This analysis inferred three distinct clusters that were the same as those identified in the STRUCTURE analysis (Fig 2). Spatial patterning indicated substantial genetic boundaries among low-, medium- and high-altitude populations.

**Fig 3:**
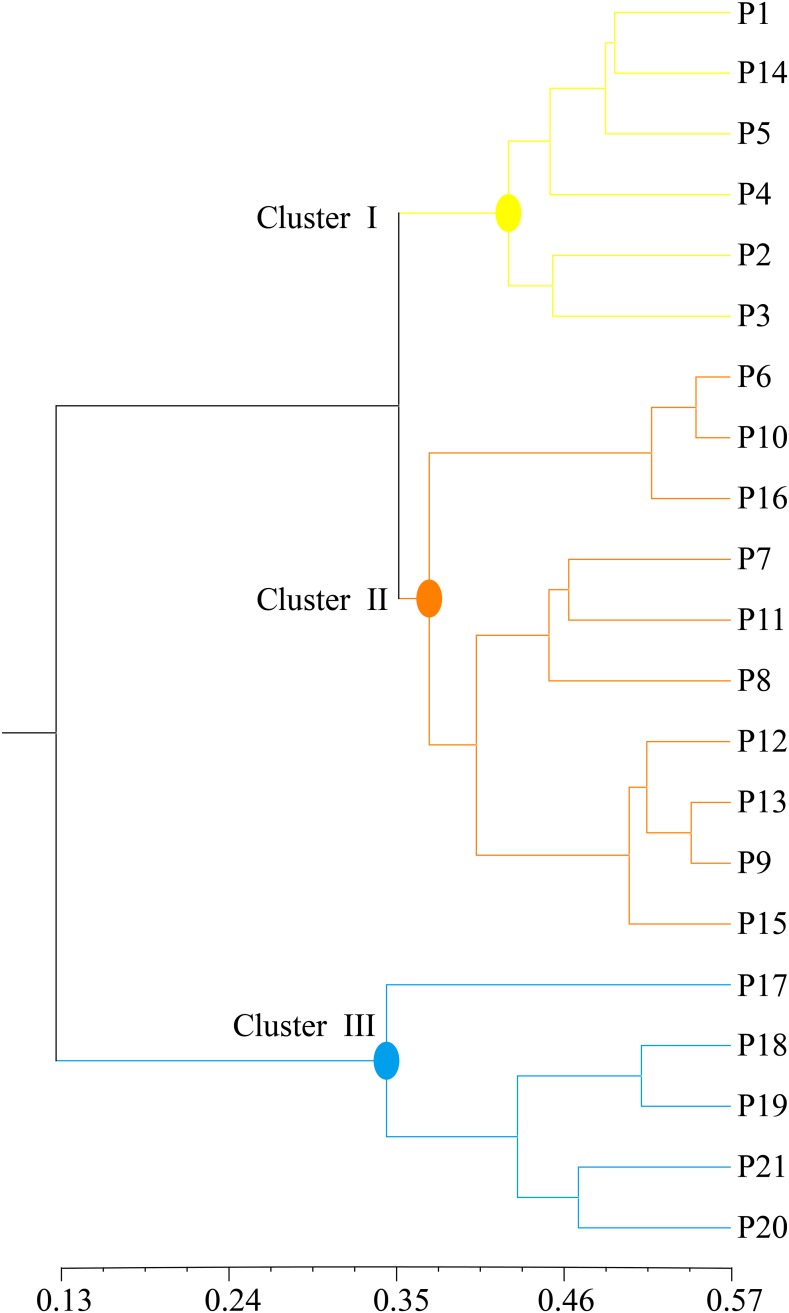
Clustering of 21 *P*. *mira* populations based on UPGMA analyses. Dendrogram of 21 populations of *P*. *mira* resulting from the UPGMA cluster analysis based on Nei’s genetic distance, which was obtained from SSR markers.

The unrooted UPGMA tree also denoted three clusters that were consistent with those of STRUCTURE and GENELAND analyses (Fig 3). Therefore, we divided the 21 *P*. *mira* populations into three genetic clusters for subsequent analyses.

**Fig 4:**
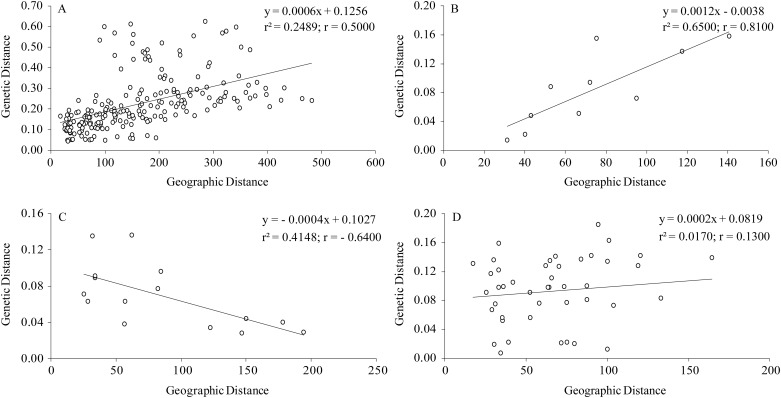
Correlation between genetic distance and geographical distance. (A) Among all populations, (B) among five populations within Cluster III, (C) among six populations within Cluster I, and (D) among 10 populations within Cluster II.

The Mantel test revealed a moderately positive correlation between genetic and geographic distance (r = 0.50, *P* = 0.01) across the entire distribution of *P*. *mira* populations (Fig 4A). This result indicated significant isolation-by-distance (IBD) among all populations. We also tested the pattern of IBD within each genetic cluster. Three separate Mantel tests indicated that a highly positive correlation between geographic and genetic distance still existed within Cluster III (r = 0.81, *P* = 0.02; Fig 4B), but it was absent within Cluster I (r = -0.64, *P* = 0.12) and Cluster II (r = 0.13, *P* = 0.19; Fig 4C and 4D).

To investigate genetic differentiation in *P*. *mira*, we conducted an AMOVA and calculated pairwise differentiation values (*F*_*ST*_) and pairwise gene flow values (*Nm*) among all populations and the three genetic clusters. Genetic differentiation between the 21 populations was significant and high as evidenced by a global *F*_*ST*_ value of 0.16 (*P* = 0.01; Table 2). The pairwise *F*_*ST*_ and pairwise *Nm* values ranged from 0.01 to 0.36 and from 0.44 to 20.33, respectively (S6 Table). Low genetic differentiation and frequent gene flow were detected between pairwise populations from the same genetic cluster. Populations in Cluster III were significantly and greatly differentiated from the populations in the other two clusters. Among the three genetic clusters, genetic differentiation was also significant and high (*F*_*ST*_ = 0.18, *P* = 0.01; Table 2). The differentiation between Clusters I and II was significant, but very slight (*F*_*ST*_ = 0.03, *P* = 0.01), whereas the genetic differentiation between Cluster III and the other clusters was significant and slightly high (*F*_*ST*_ = 0.14, *P* = 0.01 and *F*_*ST*_ = 0.13, *P* = 0.01; S7 Table). Furthermore, an AMOVA based on the 21 populations revealed 83.7% of variation within populations, and that based on the three genetic clusters detected 81.7% of variation within populations, 13.6% among the three clusters, and 4.7% among the populations within each cluster (Table 2). These results supported high genetic variation within the *P*. *mira* populations.

**Table 2:** Analysis of molecular variance (AMOVA) based on twenty-five SSR markers.

Source of variation	*d*.*f*.	Sum of squares	Variance components	Percentage of variation	Fixation index
Among 21 populations					
Among populations	20	1172.81	1.30 Va	16.3	*F*_*ST*_ = 0.16
Within populations	819	5480.55	6.69 Vb	83.7	
Total	839	6653.36	7.99	100.0	
Among three genetic clusters					
Among clusters	2	610.07	1.91 Va	13.6	*F*_*ST*_ = 0.18
Among the populations within each cluster	18	562.74	0.57 Vb	4.7	*F*_*SC*_ = 0.05
Within populations	819	5480.55	6.69	81.7	*F*_*CT*_ = 0.14
Total	839	6653.36	9.17	100.0	

*F*_*ST*_: variance among the coefficients of individuals relative to the total variance. *F*_*SC*_: variance among subpopulations within groups. *F*_*CT*_: variance among groups relative to the total variance.

### Genetic diversity

The mean *A*, *Ae*, *He*, *Ho*, and *I* values of the 21 populations were 3.8, 2.5, 0.52, 0.44, and 0.95, respectively, indicating moderate genetic diversity in *P*. *mira* populations (Table 3). The highest genetic diversity was detected in P17, and the lowest diversity was observed in P18. In addition to P4, P17, and P20, the remaining 18 populations had private alleles (*Np*), which ranged from one to four alleles per population. Interestingly, P18 had the highest number of private alleles (*Np* = 4), although it contained the lowest genetic diversity. Genetic diversity was also estimated within each genetic cluster (Table 3). Cluster II contained the highest genetic diversity, followed by Cluster I, and Cluster III had the lowest genetic diversity.

**Table 3:** Genetic diversity estimations in all populations and the three genetic clusters.

Population ID	Sample size	*A*	*Ae*	*I*	*He*	*Ho*	*F*_*IS*_	*Np*	*HWE*
P1	20	3.3 ± 0.6	2.4 ± 0.5	0.89 ± 0.09	0.52 ± 0.05	0.43 ± 0.05	0.18 ± 0.09	1	*
P2	20	3.8 ± 0.5	2.3 ± 0.4	0.92 ± 0.11	0.52 ± 0.08	0.44 ± 0.05	0.16 ± 0.04	1	--
P3	20	3.8 ± 0.4	2.6 ± 0.3	1.00 ± 0.13	0.57 ± 0.09	0.49 ± 0.08	0.13 ± 0.06	1	--
P4	20	3.6 ± 0.5	2.2 ± 0.3	0.89 ± 0.08	0.50 ± 0.07	0.45 ± 0.03	0.11 ± 0.04	0	--
P5	20	3.5 ± 0.6	2.2 ± 0.5	0.91 ± 0.04	0.53 ± 0.05	0.44 ± 0.03	0.17 ± 0.08	2	--
P6	20	3.8 ± 0.4	2.5 ± 0.3	0.95 ± 0.09	0.53 ± 0.04	0.47 ± 0.04	0.12 ± 0.07	2	*
P7	20	3.5 ± 0.5	2.6 ± 0.4	1.00 ± 0.07	0.58 ± 0.05	0.47 ± 0.03	0.19 ± 0.07	2	*
P8	20	3.5 ± 0.6	2.3 ± 0.4	0.90 ± 0.07	0.52 ± 0.05	0.44 ± 0.04	0.15 ± 0.07	1	--
P9	20	3.6 ± 0.6	2.5 ± 0.4	0.99 ± 0.08	0.57 ± 0.05	0.43 ± 0.03	0.24 ± 0.08	2	*
P10	20	3.6 ± 0.5	2.4 ± 0.5	0.94 ± 0.07	0.54 ± 0.04	0.44 ± 0.03	0.19 ± 0.07	2	*
P11	20	4.2 ± 0.6	2.7 ± 0.4	1.05 ± 0.09	0.58 ± 0.04	0.49 ± 0.04	0.15 ± 0.05	2	--
P12	20	4.1 ± 0.7	2.6 ± 0.5	1.03 ± 0.08	0.57 ± 0.04	0.42 ± 0.04	0.26 ± 0.05	2	*
P13	20	4.2 ± 0.6	2.5 ± 0.4	0.99 ± 0.08	0.54 ± 0.04	0.45 ± 0.04	0.16 ± 0.06	2	*
P14	20	4.3 ± 0.7	2.6 ± 0.5	1.03 ± 0.08	0.57 ± 0.04	0.46 ± 0.04	0.19 ± 0.05	1	--
P15	20	4.1 ± 0.7	2.5 ± 0.5	1.00 ± 0.08	0.56 ± 0.03	0.42 ± 0.05	0.25 ± 0.04	1	*
P16	20	3.9 ± 0.7	2.4 ± 0.4	0.94 ± 0.09	0.53 ± 0.04	0.42 ± 0.04	0.20 ± 0.06	1	--
P17	20	6.7 ± 0.5	4.7 ± 0.6	1.57 ± 0.05	0.63 ± 0.02	0.56 ± 0.01	0.11 ± 0.03	0	--
P18	20	2.1 ± 0.4	1.7 ± 0.5	0.49 ± 0.09	0.30 ± 0.05	0.22 ± 0.05	0.26 ± 0.10	4	*
P19	20	2.5 ± 0.4	1.8 ± 0.4	0.58 ± 0.09	0.34 ± 0.05	0.32 ± 0.05	0.06 ± 0.10	3	*
P20	20	3.6 ± 0.4	2.3 ± 0.5	0.99 ± 0.07	0.52 ± 0.04	0.43 ± 0.04	0.17 ± 0.03	0	--
P21	20	4.9 ± 0.4	2.3 ± 0.4	1.01 ± 0.07	0.51 ± 0.04	0.47 ± 0.03	0.08 ± 0.03	2	--
Mean	20	3.8 ± 0.5	2.5 ± 0.4	0.95 ± 0.08	0.52 ± 0.05	0.44 ± 0.04	0.17 ± 0.06	1.50	--
Cluster I	120	5.7 ± 0.6	2.6 ± 0.4	1.06 ± 0.09	0.56 ± 0.05	0.48 ± 0.05	0.15 ± 0.06	5	--
Cluster II	200	6.0 ± 0.5	2.8 ± 0.6	1.13 ± 0.07	0.59 ± 0.04	0.49 ± 0.03	0.16 ± 0.10	6	--
Cluster III	100	8.3 ± 0.6	2.5 ± 0.5	1.03 ± 0.06	0.53 ± 0.04	0.47 ± 0.04	0.12 ± 0.09	7	--

*A*: Number of alleles; *Ae*: Number of effective alleles; *I*: Shannon’s information index; *He*: Expected heterozygosity; *Ho*: Observed heterozygosity; *F*_*IS*_: Inbreeding coefficient; *Np*: Number of private alleles. *HWE*: Hardy-Weinberg Equilibrium;

*P < 0.05 significant; -- non-significant.

The inbreeding coefficient (*F*_*IS*_) ranged from 0.06 in P19 to 0.26 in P12 and P18, with an average of 0.17 (Table 3). The expected heterozygosity was higher than the observed heterozygosity within each population. These results indicated moderate inbreeding within *P*. *mira* populations. In addition, 10 of the 21 populations (P1, P6, P7, P9, P10, P12, P13, P15, P18, and P19) significantly deviated from the *HWE*.

### Morphological clustering and variation

We measured and calculated 11 morphological characteristics of 8400 fruits and 8400 nutlets from the 21 populations, and we used these morphological data to analyze morphological variation among and within *P*. *mira* populations. An unrooted UPGMA tree was constructed using the dataset based on the 11 morphological characteristics. Similar to the results based on SSR markers, the 21 populations were assigned to three distinct clusters according to their altitudes (S3 Fig).

**Fig 5:**
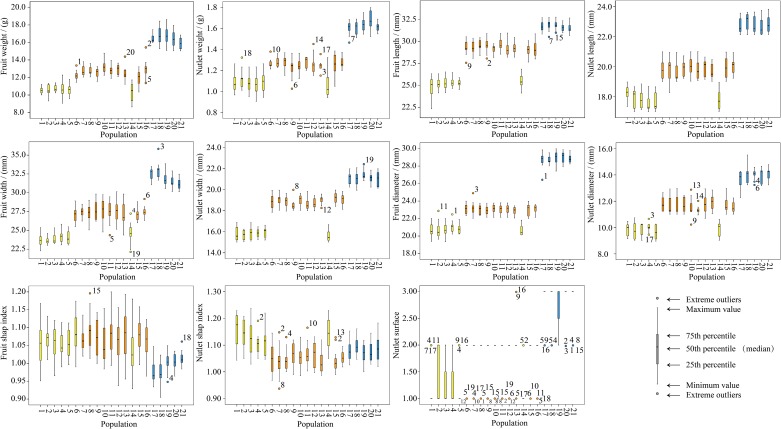
Boxplots based on 11 morphological characteristics.

When the 21 populations were divided into three clusters based on the morphological data, with the exception of the fruit shape index, the remaining 10 morphological characteristics exhibited a wide range of variation and significant differences among the different clusters (S8 Table). The nutlet surface of most individuals was smooth in Cluster I, shallow groove in Cluster II, and deep groove in Cluster III (S4 Fig, S9 Table). Most of the morphological characteristics increased as the geographic altitudes of populations increased. For instance, low-altitude populations in Cluster I had the lightest and smallest fruits and nutlets, and high-altitude populations from Cluster III contained the heaviest and largest fruits and nutlets. Furthermore, all characteristics exhibited no or low differences among the populations within each cluster (S8 Table, Fig 5). Among the six populations in Cluster I, five characteristics (fruit weight, fruit diameter, nutlet width, nutlet diameter, and nutlet surface) exhibited no significant differences, and the remaining showed low morphological variations. Among the 10 populations from Cluster II, no significant variation was detected for four characteristics (fruit width, fruit diameter, nutlet weight, and nutlet surface), but a low degree of differences was observed for seven characteristics. In Cluster III, only four characteristics (fruit width, fruit shape index, nutlet shape index, and nutlet diameter) indicated low differences among the five populations.

The coefficients of variation (*VC*) within populations ranged from 1.30% to 36.17% (S8 Table). The *F* values among populations were higher than those within populations for all morphological characteristics. These results indicated that most of the variation occurred among populations, which was not consistent with our molecular analysis results.

## Discussion

Knowledge of genetic diversity and morphological variation can provide basic information that can aid our understanding of plant evolutionary history and the protection of extant wild resources. In this study, we sampled 420 *P*. *mira* individuals from 21 wild populations to study their genetic diversity, population structure, and morphological variation by conducting molecular analyses and morphological evaluations.

### Population structure and differentiation

The STRUCTURE and GENELAND analyses based on SSR markers revealed three genetic clusters, which were highly correlated with geographic altitudes. The most likely reason for this result is that altitude differences led to significant climatic and environmental differences in water, soil, light, and temperature among the populations distributed at different altitude ranges. Climatic and environmental factors can significantly influence plant geographic distributions, evolutionary patterns, and phenological phases [45]. According to the results of our field investigation, *P*. *mira* populations at high altitudes usually flower five to 14 days later than populations at low altitudes. This phenomenon can significantly reduce the gene flow through pollen, and it can shape clear structures among populations that were distributed at different altitudinal ranges.

Mantel et al. [46] suggested that large geographic distances would limit pollen and seed dispersal among populations. In this study, moderately positive IBD was detected among all *P*. *mira* populations. However, although we conducted separate Mantel tests within clusters, different results were revealed. All of these results indicated that geographic distance is not a crucial factor for the current genetic composition in partial small-scale areas. Regarding populations from Clusters I and II, we inferred that altitude gradients might have more significant effects on population structure than geographic distance. For instance, P14 was not assigned to Cluster II based on pairwise geographic distances, but was instead grouped with Cluster I because of its geographic altitude.

Our findings indicated that Cluster III was significantly and highly differentiated from the other two clusters. Five populations from Cluster III and 16 populations from Cluster I and Cluster II are distributed in Shannan and Linzhi, respectively. In the central region of the Tibet plateau, numerous mountains, rivers, and valleys shape geographic barriers between the two geographic groups, thereby leading to the observed high genetic differentiation. However, low gene flow values (*Nm* < 1) were observed between pairwise populations from different geographic regions, thus indicating that the gene flow cannot combat genetic differentiation caused by genetic drift [16, 18]. According to Slaktin et al. [47], the founder effect is an important form of genetic drift in isolated populations, so the genetic drift caused by “founder effects” might have affected the genetic structures of *P*. *mira* populations.

### Genetic diversity of populations

Molecular analyses revealed moderate genetic diversity (*A* = 3.8, *Ae* = 2.5, *Ho* = 0.44, *He* = 0.52, *I* = 0.95) in *P*. *mira* populations. The genetic diversity of *P*. *mira* in this study was higher than that previously reported for *P*. *mira* (*A* = 1.7, *Ae* = 1.4, *I* = 0.35) [48] and other related peach species (*A* = 2.7, *Ae* = 2.1, *Ho* = 0.32–0.34, *He* = 0.23–0.46) [49,50]. Theoretically, the genetic diversity of endangered species should be low, but this was not the case for *P*. *mira*. Moderate or even high levels of genetic diversity have been reported in some endangered species [51,52]. Regarding *P*. *mira*, high adaption to various environments and wide distributions might have helped shape and preserve the genetic diversity of populations under poor living conditions [53,54]. For example, more than 60% of Tibetan areas are mountains, and the impact of topography and elevation led to significant climatic and environmental differences in water, soil, light, and temperature, thus resulting in a number of distinct and natural geographical areas. Overall, the natural ecological conditions of the Tibet region are poor because the plateau climate is characterized by thin air, low air pressure, reduced oxygen, thin soil, low rainfall, stronger solar radiation, perennial ice and snow, and frost weathering [55]. The effective population size of *P*. *mira* is relatively large, but more recent periods of excessive logging and fragmentation due to habitat destruction may have led to the initial loss of genetic diversity, despite the short period of fragmentation. Therefore, this factor might be also responsible for the moderate genetic diversity in *P*. *mira*.

Positive inbreeding coefficients were detected within each population and at each locus, indicating the significant excess of homozygotes in *P*. *mira*. Deforestation and geographic isolation have led to the heavy fragmentation of *P*. *mira* habitats [6,48,55]. Habitat fragmentation can limit genetic exchange among populations, and it can increase inbreeding within a population [56–58]. Thus, moderate inbreeding in *P*. *mira* could be attributed to habitat fragmentation. Previous studies [55] revealed that human activities such as over-harvesting and deforestation had significant effects on wild *P*. *mira* resources near rural areas in the Tibetan plateau. In this study, we found 7 populations near rural areas that deviated from *HWE*, thus indicating that recent human activities might have significant effects on these *P*. *mira* populations.

### Genetic and morphological variation

Perennial woody plant species are expected to preserve high variation within populations because of their long life cycles [59–61]. An AMOVA based on SSR markers revealed that most of the total genetic variation (83.7% and 81.7%) occurred within *P*. *mira* populations, which is higher than that reported previously based on SSR (81.0%), ISSR (71.0%), and AFLP (74.48%) data [7,48]. This discrepancy can be attributed to the fact that the number of individuals analyzed in this study was considerably greater than that in previous studies. For *P*. *mira*, an outcrossing system and long life cycle (over 1000 years) can improve gene flow and preserve shared ancestral genetic information among populations, thus significantly improving genetic variation within populations.

Both molecular and morphological data revealed three clusters that were correlated with geographic altitudes. Interestingly, our morphological analyses showed fewer differences within populations, and this was contrary to our molecular analysis results. The morphological variation among the three clusters was higher than that within clusters. *P*. *mira* individuals are distributed over a narrow altitude range within each population, although they are widely distributed in the Tibet plateau. Combined with our molecular results, we concluded that the morphological differences may be caused by the relatively isolated distributions and various climates and environments associated with *P*. *mira* populations from different geographic altitudes. For instance, high-altitude populations contained larger and heavier fruits and nutlets than the medium- and low- altitude populations. These results were consistent with the findings of Pluess et al. [62] in that large seeds could be retained at high-altitude areas because of their high seedling rates under poor environmental conditions.

### Conservation strategy

Studying genetic diversity, population structure, and morphological variations could provide basic information about plant conservation [63]. Although the levels of genetic diversity of *P*. *mira* populations were moderate, some populations still exhibited high risk of genetic variation loss, and human activities and climates had significant effects on most *P*. *mira* populations. Therefore, there is also an urgent need to conduct *P*. *mira* resource conservation.

The analyses based on SSR markers and morphological characteristics revealed three completely consistent clusters among the 21 wild *P*. *mira* populations. Each cluster contained unique genetic and morphological variations and had different evolutionary histories. Therefore, the conservation efforts should be divided into three units that correspond to the three genetic and morphological clusters. Because the six populations in Cluster I were found in national nature reserves, more attention needs to be paid to the other clusters. Furthermore, *in situ* conservation should prioritize populations with high genetic diversity in Clusters II and III such as P11 and P17. Because human activities have had significant effects on many *P*. *mira* populations (P6, P7, P9, P10, P12, P13, and P15), we propose the strengthening of conservation awareness in local farmers and the hanging of placards to reduce deforestation and prevent continuing habitat deterioration. We also suggest *ex situ* conservation for individuals with unique traits and populations with high inbreeding to prevent the potential loss of genetic variation. Furthermore, improving the genetic diversity within populations requires the monitoring of crossing among populations.

## Conclusions

This study comprehensively analyzed the genetic diversity, population structure, and morphological variation of 21 wild *P*. *mira* populations in the Tibet plateau. Moderate levels of genetic diversity were detected in *P*. *mira* populations. Significant homozygote excess was observed within each population, and this might be the result of sibling mating caused by habitat fragmentation. Furthermore, human activities might be responsible for the deviation of numerous *P*. *mira* populations from the *HWE*. Both molecular and morphological analyses assigned the 21 populations to three clusters, which were significantly correlated with the geographic altitudes. Because of the presence of geographic barriers and genetic drift, populations in Shannan were highly differentiated from populations in Linzhi. Lastly, we formed three conservation units and proposed several conservation strategies for these studied populations.

## Supporting information

S1 Fig Geographic distribution of the analyzed *Prunus mira* Koehne samples from Tibet. (TIF)

S2 Fig Determination of the optimal number (K) of subpopulations for *P*. *mira* populations based on STRUCTURE. (TIF)

S3 Fig Unrooted UPGMA tree constructed using 11 morphological characteristics. (TIF)

S4 Fig The morphological characteristics of nutlets and nutlet surfaces associated with the 21 *P*. *mira* populations. The nutlet surfaces of 420 *P*. *mira* individuals can be divided into three clusters based on visual inspection. The first cluster was smooth (P1–P5, P14), the second cluster exhibited nutlet surfaces with shallow grooves (P6, P7, P8, P9, P10, P11, P12, P13, P15, and P16), and the third cluster displayed nutlet surfaces with deep grooves (P17, P18, P19, P20, and P21).
(TIF)

S1 Table Primer information for 25 microsatellite loci used to analyze 420 *P*. *mira* individuals. (XLSX)

S2 Table List of the 420 individuals studied and their alleles at each SSR. (XLSX)

S3 Table Diversity indices of the 25 nuclear microsatellite loci from the data of 420 *P*. *mira* individuals. (XLSX)

S4 Table Mean ancestry values for the 21 *P*. *mira* populations at K = 2 and K = 3 inferred by STRUCTURE. (XLS)

S5 Table Mean ancestry values for the 420 *P*. *mira* individuals at K = 2 and K = 3 inferred by STRUCTURE. (XLS)

S6 Table Genetic differentiation and gene flow estimated using pairwise *F*_*ST*_ (below diagonal) and *Nm* (above diagonal) among the 21 populations. (XLSX)

S7 Table Genetic differentiation and gene flow estimated using pairwise *F*_*ST*_ (below diagonal) and *Nm* (above diagonal) among the three genetic clusters. (XLSX)

S8 Table Duncan’s multiple comparison analysis and variation coefficients (*CV*) of fruit and nutlet morphological characteristics among and within *P*. *mira* populations. (XLSX)

S9 Table Data associated with 11 morphological characteristics of the 420 *P*. *mira* individuals. (XLSX)
