# Supplementary figures and images for: Genetic diversity and population structure of Prunus mira (Koehne) from the Tibet plateau in China and recommended conservation strategies
Source: PLoS One. 2017 Nov 29;12(11):e0188685. doi: 10.1371/journal.pone.0188685 (PMC5706700; doi:10.1371/journal.pone.0188685)

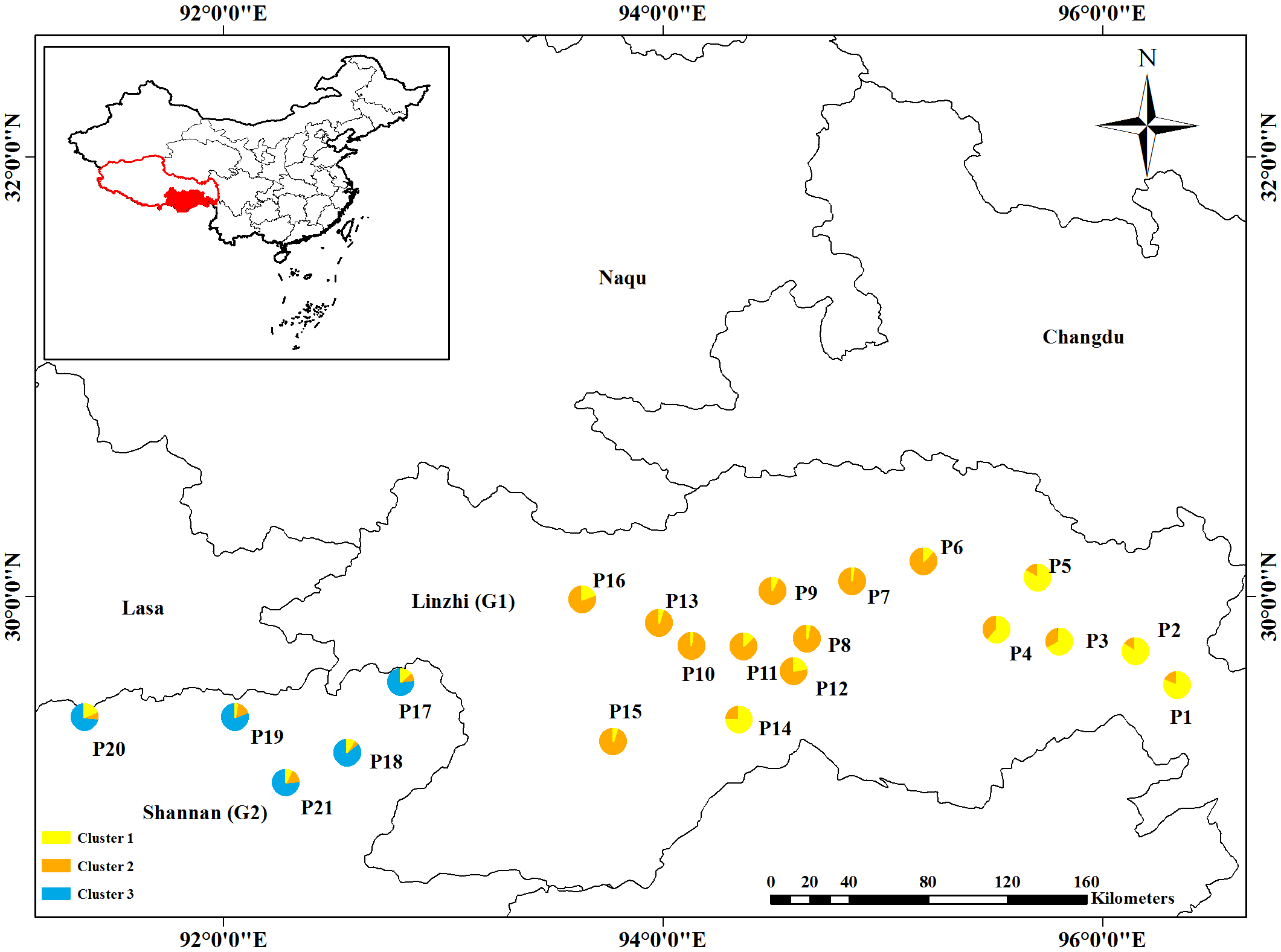

Supplement: S1 Fig — (TIF) [file pone.0188685.s001.tif]

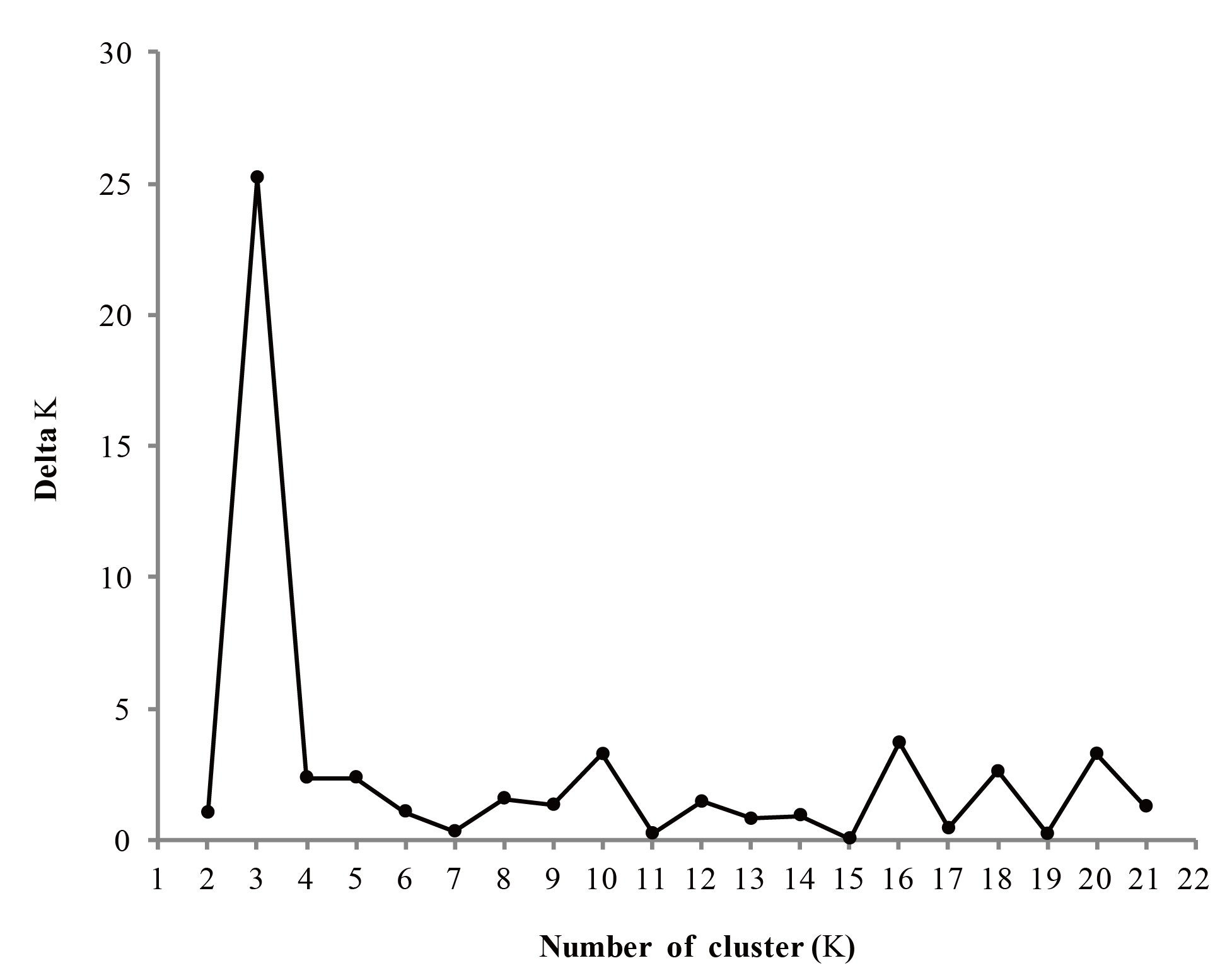

Supplement: S2 Fig — (TIF) [file pone.0188685.s002.tif]

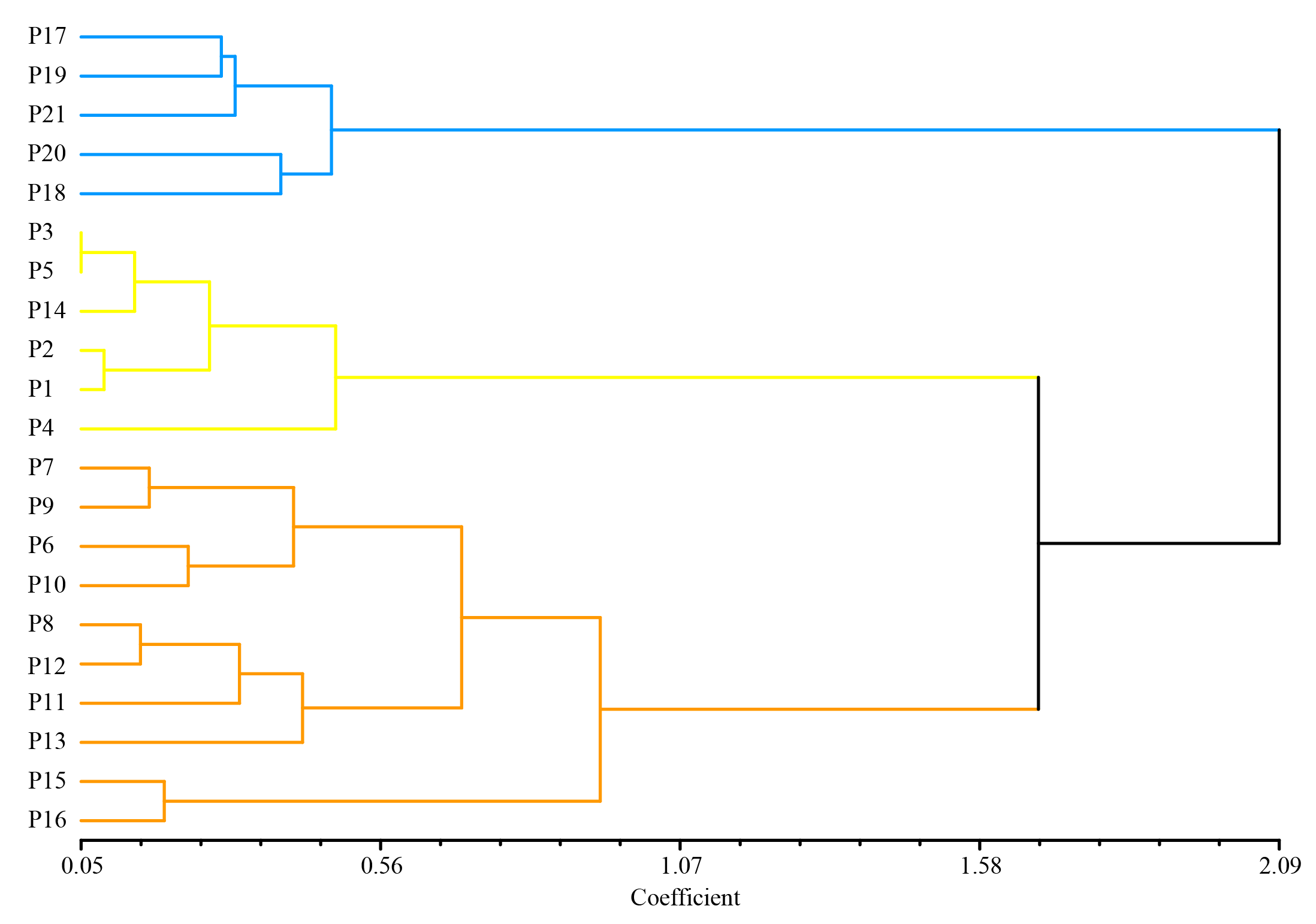

Supplement: S3 Fig — (TIF) [file pone.0188685.s003.tif]

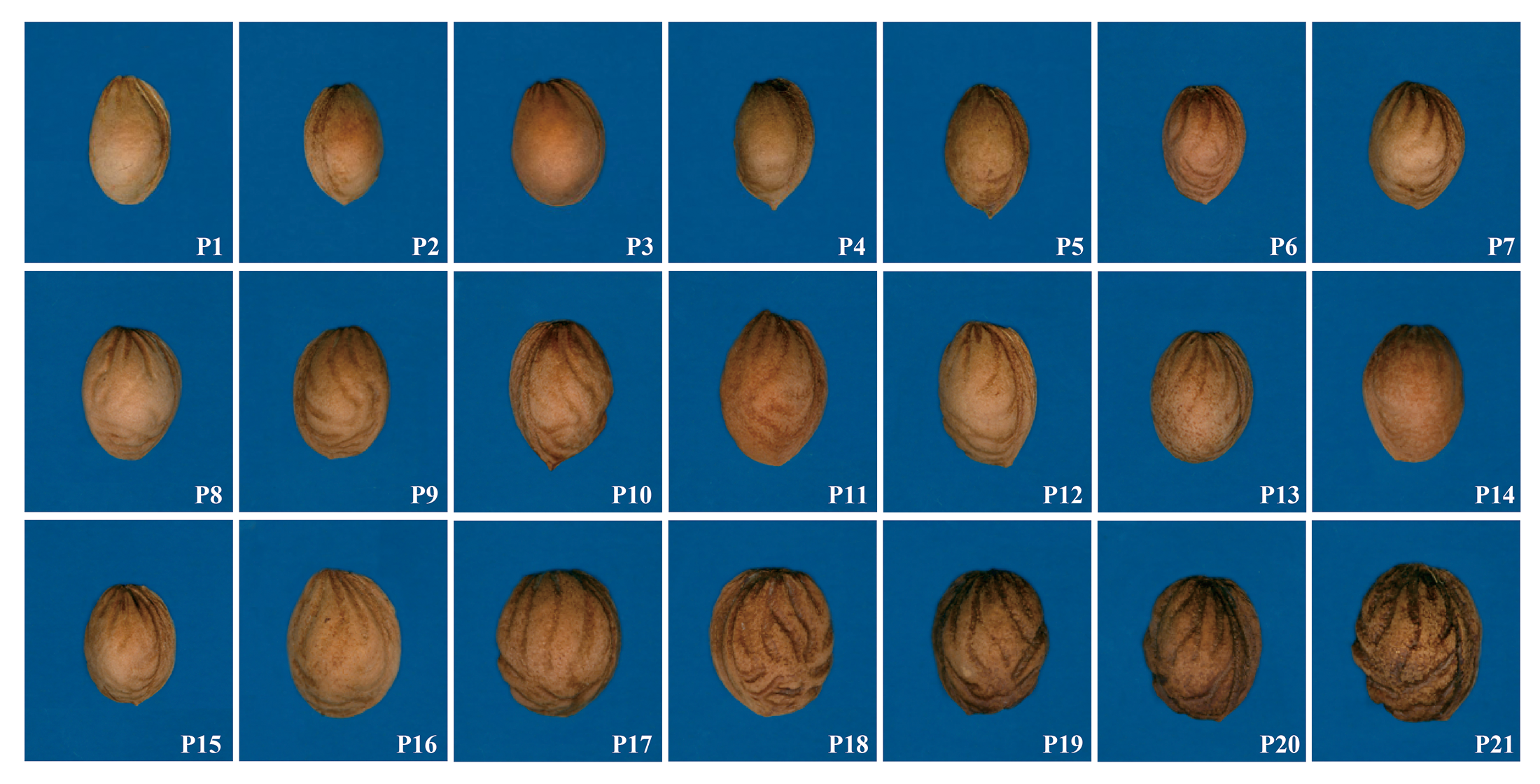

Supplement: S4 Fig — The nutlet surfaces of 420 P. mira individuals can be divided into three clusters based on visual inspection. The first cluster was smooth (P1–P5, P14), the second cluster exhibited nutlet surfaces with shallow grooves (P6, P7, P8, P9, P10, P11, P12, P13, P15, and P16), and the third cluster displayed nutlet surfaces with deep grooves (P17, P18, P19, P20, and P21). (TIF) [file pone.0188685.s004.tif]
